# Synthesis of 1-(4-Trifluoromethoxyphenyl)-2,5-dimethyl-3-(2-*R*-thiazol-4-yl)-1*H*-pyrroles *via* Chain Heterocyclization

**DOI:** 10.3390/molecules15020997

**Published:** 2010-02-23

**Authors:** Mykhaylo V. Vovk, Oleksandr M. Pinchuk, Andrij O. Tolmachov, Andrei A. Gakh

**Affiliations:** 1 Institute of Organic Chemistry, NAS of Ukraine, Murmanska 5, 02094 Kyiv, Ukraine; 2 National Taras Shevchenko University, Volodymyrs`ka 62, 01033 Kyiv, Ukraine; 3 Oak Ridge National Laboratory, Oak Ridge, TN 37831-6242, USA

**Keywords:** pyrrole, fluorinated heterocycles, Paal-Knorr reaction, trifluoromethoxy group, chain heterocyclization

## Abstract

The title compounds, (4-trifluoromethoxyphenyl)-2,5-dimethyl-3-(2-*R*-thiazol-4-yl)-1*H*-pyrroles, were prepared in four steps starting from commercially available 4-trifluoromethoxyaniline. The pyrrole (second ring) was added in one step using the Paal-Knorr method. The thiazole (third ring) was added in three steps using chloroacylation with chloroacetonitrile followed by heterocyclization with thioamides/thioureas.

## 1. Introduction

In the course of our search for new anti-cancer compounds that can be used in chemotherapy of late androgen-independent stages of prostate cancer, we turned our attention to the specific class of fluoroheterocyclic systems containing three linked rings, A-B-C, where B is a heterocyclic ring, and A and C are either a heterocyclic or an aromatic ring. Typically, these heterocyclic systems are produced by connecting of one heterocyclic and two aromatic or heterocyclic fragments together using appropriate linking methods. Unfortunately, many common linking procedures cannot be used for direct ring connections. An alternative approach entails chain heterocyclization where heterocyclic moieties are being added in one-by-one fashion using appropriate alicyclic components. Here we report the results of successful application of this strategy for the synthesis of A-B-C fluoroheterocyclic systems with pyrrole as the central unit B.

The pyrrole ring is a part of many natural compounds [1,2,3] as well as synthetic biologically active molecules [4,5]. For example, dialkylaminoalkyl esters of *N*-(4-hydroxyphenyl)pyrrole exhibit anesthetic and hypotensive properties [6], *N*-(3-carboxyphenyl)pyrroles are known as antiphlogistic compounds [7], and *N*-(4-phenoxyphenyl)pyrroles have anticholesteremic properties [8]. Some derivatives of 4-(2,5-dimethylpyrrolyl)benzoic acid inhibit Anthrax Lethal Factor (LF) [9] and Serotonin *N*-Acetyltransferase [10]. The trifluoromethoxy group is known for its utility in synthesis [11] and usefulness in the optimization of biologically active compounds [12,13,14,15]. Moreover, heterocyclic systems bearing the СF_3_O group are especially attractive, given their presence in, among others, the insecticide indoxacarb16], the acaricide flufenerim [17], the plant growth regulator flurprimidol [18], and the neurologic drug riluzole [19]. At the same time, little is known about heterocyclic systems containing CF_3_O-group(s) and pyrrole rings. Our research in this area started with the synthesis of 1-(4-trifluoromethoxyphenyl)-2,5-dimethyl-3-chloroacetylpyrrole - a new building block for the synthesis of extended heterocyclic libraries containing the 1-(4-trifluoromethoxyphenyl)pyrrole fragment [20].

## 2. Results and Discussion

The Paal-Knorr method [21] (which entails condensation of 1,4-diketones with anilines) is the most frequently used method for the synthesis of 1-aryl-2,5-dialkyl(aryl) substituted pyrroles. For example, 1-(4-trifluoromethoxyphenyl)-2,5-dimethylpyrrole (**3**) can be prepared in 76% yield from readily available 2,5-hexanedione (**1**) and 4-trifluoromethoxyaniline (**2**) by this route. According to the literature data [22,23], Friedel-Crafts acylation of 1-phenyl-2,5-pyrrole with alkylcarboxylic acid chlorides yields bis-acylated products (3,4-diketones), whereas acylation with aryl carboxylic acid chlorides results in a mixture of mono- (3-acylated) and bis- (3,4-acylated) products. It appears that direct acylation with chloroacetyl acid chloride cannot be used for selective preparation of the desired 3-chloroacetyl-2,5-dimethylpyrrole (**5**).

**Scheme 1:**
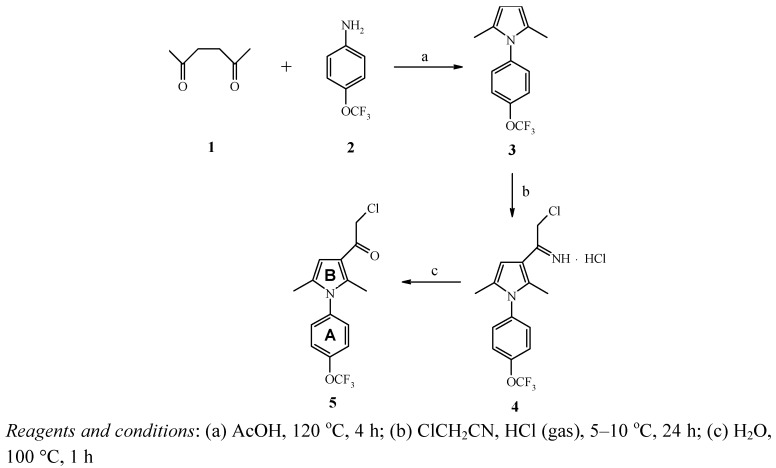
Synthesis of 3-chloroacetylpyrrole (**5**).

**Scheme 2:**
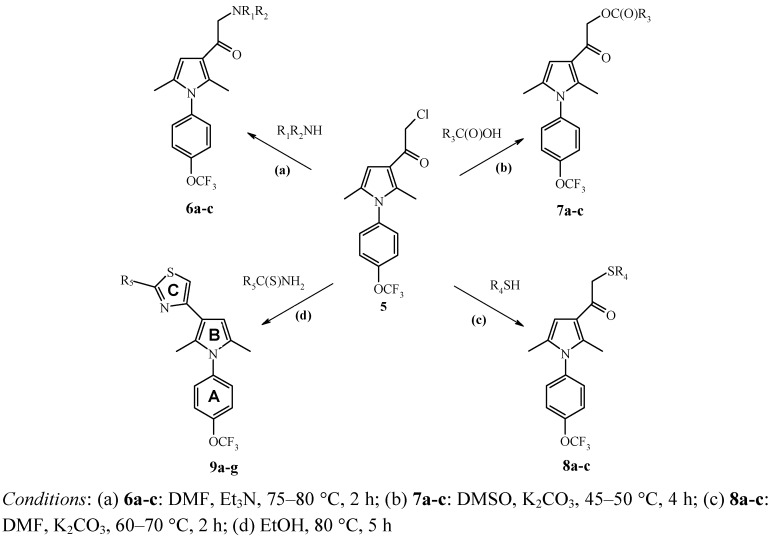
Synthesis of 4-trifluoromethoxyphenyl substituted pyrroles **6**-**9** (R_1_-R_5_: see Table 1).

We developed a new preparative method for the synthesis of 3-chloroacetylpyrroles using mild and selective chloroacetylimidoyl chloride [24] that can be prepared *in situ* from chloroacetonitrile and hydrogen chloride. The desired iminium salt precipitates directly from the reaction mixture in 87% yield. Subsequent hydrolysis gives the target product **5** in 69 % yield (Scheme 1). The presence of a reactive chloroacetyl group in pyrrole **5** makes this compound an attractive building block for the preparation of various nitrogen-, oxygen- and sulfur-containing compounds containing a 4-trifluoromethoxyphenyl fragment. For example, *N*-alkylation of imidazole, isothiazole, tetrazole derivatives; O-alkylation of hetarylcarboxylic and hetarylacetic acids; S-alkylation of thiazole, 1,3,4-oxadiazole and tetrazole derivatives proceed easily and in mild conditions in DMSO or DMF solutions. The use of thioamides and thioureas in reactions with chloroacetylpyrrole (**5**) allowed us to add the desired third heterocyclic ring (thiazole fragment) as the last step of chain heterocyclization. As a result, new 4-trifluoromethoxyphenyl substituted pyrroles **6a-c, 7a-c, 8a-c, 9a-g** functionalized with a set of pharmacophoric heterocyclic groups were successfully prepared using this approach (Scheme 2, Table 1).

**Table 1:** Yields and melting points of the synthesized compounds.

Product	R	Yield, %	Mp	Product	R	Yield, %	Mp
**6a**	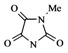 (R_1_R_2_N)	76	174-175	**8c**	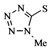 (SR_4_)	92	106
**6b**	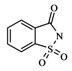 (R_1_R_2_N)	81	232-234	**9a**	Me (R_5_)	62	134-135
**6c**	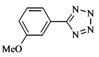 (R_1_R_2_N)	70	126-127	**9b**	2-Thienyl (R_5_)	65	115-116
**7a**	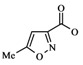 [OC(O)R_3_]	71	143-144	**9c**	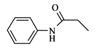 (R_5_)	69	131-132
**7b**	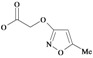 [OC(O)R_3_]	74	110-111	**9d**	NH_2_ (R_5_)	71	126-127
**7c**	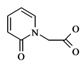 [OC(O)R_3_]	78	103	**9e**	MeNH (R_5_)	64	166-167
**8a**	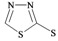 (SR_4_)	84	132-133	**9f**	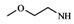 (R_5_)	70	129-130
**8b**	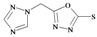 (SR_4_)	89	165-166	**9g**	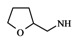 (R_5_)	65	108-109

## 3. Experimental

### 3.1. General

Melting points were determined with a Kofler micro hot-stage (Reichert, Wien) and were uncorrected. ^1^H- and ^19^F-NMR spectra were recorded in DMSO-d_6_ on a Varian-Gemini spectrometer at 299.94 and 188.14 MHz, respectively, with TMS (^1^H) and CCl_3_F (^19^F) as internal standards. ^13^C-NMR spectra were recorded on a Bruker Arano DRX-500 spectrometer (125.75 MHz), with TMS as internal standard. APCI MS data were obtained on an Agilent 1100\DAD\MSD VL G1965a instrument.

*2,5-Dimethyl-1(4-(trifluoromethoxyphenyl)-1H-pyrrole* (**3**). A mixture of 4-trifluoromethoxyaniline (30 g, 170 mmol) and 2,5-hexanedione (22.8 g, 200 mmol) in acetic acid (150 mL) was refluxed for 4 h. The reaction mixture was cooled and poured into water (500 mL), the precipitated solid was filtered and dried. Yield 32.9 g (76 %); mp 68 °C; ^1^H-NMR: δ 1.98 (s, 6H), 5.76 (s, 2H), 7.34 (d, 2H, *J* = 9.0 Hz), 7.41 (d, 2H, *J* = 9.0 Hz); ^13^C-NMR: δ 12.66 (2 CH_3_), 106.18 (pyrrole, 3,4-C), 120.57 (q, CF_3_, *J* = 254.6 Hz), 121.64 (4-CF_3_O-C_6_H_4_, 3,5-C), 127.60 (pyrrole, 2,5-C), 129.90 (4-CF_3_O-C_6_H_4_, 2,6-C), 137.32 (4-CF_3_O-C_6_H_4_, 1-C), 147.33 (4-CF_3_O-C_6_H_4_, 4-C); ^19^F-NMR: δ -57.75 (F_3_CO); MS: *m/z* 256.0 (M+1)^+^; Anal. Calcd. for C_13_H_12_CF_3_NO: C 61.18; H 4.74; N 5.49. Found: C 61.31; H 4.77; N 5.61.

*2-Chloro-1-{2,5-dimethyl-1-[(4-trifluoromethoxy)phenyl]-1H-pyrrol-3yl}-ethanimine hydrochloride* (**4**). Dry hydrogen chloridewas bubbled through a solution of 2,5-dimethyl-1[4-(trifluoromethoxy)phenyl]-1*H*-pyrrole (32.9 g, 130 mmol ) and chloroacetonitrile (15 g, 200 mmol) in diethyl ether (200 mL) for 2 hours with vigorous stirring at 5-10 ^о^С. The reaction mixture was left for 12 hours at room temperature. The precipitated solid was collected by filtration, washed with diethyl ether and dried in air. Yield 41.4 g (87 %); mp 220^o ^C; ^1^H-NMR: δ 2.23 (s, 3H), 2.36 (s, 3H), 5.11 (s, 2H), 7.015 (s, 1H), 7.44 (d, 2H, *J* = 9.0 Hz), 7.51 (d, 2H, *J =* 9.0 Hz), 11.62 (s, 1H), 11.96 (s, 1H); ^19^F-NMR: δ - 57.14 (F_3_CO); Anal. Calcd. for C_15_H_15_Cl_2_F_3_NO_2_: C 49.07; H 4.19; Cl 19.31. Found: C 49.23; H 4.08; Cl 19.17.

*2-Chloro-1-{2,5-dimethyl-1-[4-(trifluoromethoxy)phenyl]-1H-pyrrol-3-yl}-ethanone* (**5**). A suspension of 2-chloro-1-{2,5-dimethyl-1-[(4-trifluoromethoxy)phenyl]-1*H*-pyrrol-3yl}-ethanimine hydrochloride (**4**, 41.4 g, 110 mmol) in water (200 mL) was heated under reflux for 1 h. The reaction mixture was cooled to room temperature; the precipitated solid was collected, washed with water, and dried. The product was crystallized from methanol. Yield 25.8 g (69 %); mp 147 ^o^C;IR (KBr, cm^-1^) 1685; ^1^H- NMR: δ 1.94 (s, 3H), 2.22 (s, 3H), 4.75 (s, 2H), 6.48 (s, 1H), 7.49 (d, 2H, *J =* 9.0 Hz), 7.56 (d, 2H, *J =* 9.0 Hz); ^13^C-NMR: δ 12.28 (CH_3_), 12.49 (CH_3_), 47.80 (CH_2_), 107.40 (pyrrole, 4-C), 117.04 (pyrrole, C-5), 121.45 (q, CF_3_, *J =*257.8 Hz), 123.19 (4-CF_3_O-C_6_H_4_, 3,5-C), 128.73 (pyrrole, 3-C), 130.02 (4-CF_3_O-C_6_H_4_, 2,6-C), 135.35 (4-CF_3_O-C_6_H_4_, 1-C), 136.09 (pyrrole, 2-C), 148.15 (q, 4-CF_3_O-C_6_H_4 _, 4-C, *J =* 1.3 Hz), 186.80 (C=O); ^19^F-NMR: δ -56.89 (F_3_CO); MS: *m/z* 332.0 (M+1)^+^; Anal. Calcd. for C_15_H_13_ClF_3_NO_2_: C 54.31; H 3.95; N 4.22. Found: C 54.20; H 3.87; N 4.11.

### 3.2. General method for synthesis of 2,5-dimethyl-3-(2-N-substituted-1-oxoethyl)-1-[4-(trifluoro-methoxy)phenyl]-1H-pyrroles **6a-c**

An appropriate heterocyclic compound (5 mmol) and triethylamine (1.0 mL, 7.5 mmol) were added to a solution of 2-сhloro-1-{2,5-dimethyl-1-[4-(trifluoromethoxy)phenyl]-1*H*-pyrrol-3-yl}-ethanone (**5**, 1.66 g, 5 mmol) in DMF (50 mL). The reaction mixture was heated at 75-80 °C with stirring for 2 h, and then it was cooled and poured into water (100 mL). The precipitated solid was filtrated and crystallized from dioxane.

*1-{2-[2,5-Dimethyl-1(4-trifluoromethoxyphenyl)-1H-pyrrol-3-yl]-2-oxoethyl}-3-methylinidazolidine-2,4,5-trione* (**6a**). ^1^H-NMR: δ 1.99 (s, 3H), 2.23 (s, 3H), 3.07 (s, 3H), 4.81 (s, 2H), 6.63 (s, 1H), 7.48 (d, 2H, *J =* 9.0 Hz), 7.54 (d, 2H, *J =* 9.0 Hz); ^19^F-NMR: δ - 57.47 (F_3_CO); MS: *m/z* 424.0 (M+1)^+^; Anal. Calcd. for C_19_H_16_F_3_N_3_O_5_: C 55.93; H 3.81; N 9.93. Found: C 56.04; H 3.88; N 9.80.

*2-{2-[2,5-Dimethyl-1-(4-trifluoromethoxyphenyl)-1H-pyrrol-3-yl]-2-oxoethyl}-1,1-dioxo-1,2-dihydro-1λ^6^-benzo[d]isothiazol-3-one* (**6b**). ^1^H-NMR: δ 2.01 (s, 3H), 2.24 (s, 3H), 4.97 (s, 2H), 6.67 (s, 1H), 7.47 (d, 2H, *J =* 7.7 Hz), 7.53 (d, 2H, *J =* 8.8 Hz), 8.05 (m, 3H), 8.38 (d, 1H, *J =* 8.8 Hz); ^19^F-NMR: δ ‑ 57.44 (F_3_CO). MS: m/z 479.0 (M+1)^+^; Anal. Calcd. for C_22_H_17_F_3_N_2_O_5_S: C 55.23; H 3.58; N 5.86. Found: C 55.35; H 3.68; N 5.97.

*1-[2,5-Dimethyl-1-(4-trifluoromethoxyphenyl)-1H-pyrrol-3-yl]-2-[5-(3-methoxyphenyl)-tetrazol-2-yl]-ethanone*** (6c)**. ^1^H-NMR: δ 2.02 (s, 3H), 2.25 (s, 3H), 3.86 (s, 3H), 6.19 (s, 2H), 6.65 (s, 1H), 7.09 (dd, 1H, *J =* 7.4 Hz, *J =* 2.3 Hz), 7.59 (m, 7H). ^19^F-NMR: δ - 57.49 (F_3_CO); MS: *m/z* 472.0 (M+1)^+^; Anal. Calcd. for C_23_H_20_F_3_N_5_O_3_: C 58.60; H 4.28; N 14.86. Found: C 58.93; H 4.09; N 14.64.

### 3.3. General method for synthesis of2,5-dimethyl-3-(2-O-substituted 1-oxoethyl)-1-[4-(trifluoromethoxy)-phenyl]-1H-pyrroles **7 a-c**

An appropriate acid (5 mmol) and potassium сarbonate (1.4 g, 10 mmol) were added to a solution of 2-сhloro-1-{2,5-dimethyl-1-[4-(trifluoromethoxy)phenyl]-1H-pyrrol-3-yl}-ethanone (**5**, 1.66 g, 5 mmol) in DMSO (50 mL). The reaction mixture was heated at 45-50^о^С with stirring for 4 h. The reaction mixture was cooled and poured into water (100 mL). The precipitated solid was collected, dried and recrystallized from 2-propanol.

*3-Methylisoxazole-5-carboxylic acid 2-[2,5-dimethyl-1(4-trifluoromethoxyphenyl)-1H-pyrrol-3-yl]-2-oxoethyl ester* (**7a**). IR (KBr, cm^-1^) 1700, 1775; ^1^H-NMR: δ 1.99 (s, 3H), 2.25 (s, 3H), 2.36 (s, 3H), 5.41 (s, 2H), 6.50 (s, 1H), 7.21 (s, 1H), 7.49 (d, 2H, *J =* 9.0 Hz), 7.54 (d, 2H, *J =* 9.0 Hz); ^19^F-NMR: - 57.47 (F_3_CO); MS: *m/z* 423.0 (M+1)^+^; Anal. Calcd. for C_20_H_17_F_3_N_2_O_5_: C 56.88; H 4.06; N 6.63. Found: C 56.79; H 4.10; N 6.59.

*(5-Methylisoxazol-3-yloxy)acetic acid 2-[2,5-dimethyl-1-(4-trifluoromethoxyphenyl)-1H-pyrrol-3-yl]-2-oxoethyl ester* (**7b**). IR (KBr, cm^-1^) 1695, 1770; ^1^H-NMR: δ 1.97 (s, 3H), 2.24 (s, 3H), 2.33 (s, 3H), 4.95 (s, 2H), 5.21 (s, 2H), 6.03 (s, 1H), 6.46 (s, 1H), 7.47 (d, 2H, *J =* 8.7 Hz), 7.52 (d, 2H, *J =* 8.7 Hz); ^19^F-NMR: - 57.50 (F_3_CO); MS: *m/z* 453.0 (M+1)^+^; Anal. Calcd. for C_21_H_19_F_3_N_2_O_6_: C 55.76; H 4.23; N 6.19. Found: C 55.65; H 4.24; N 6.12.

*(2-Oxo-2H-pyridin-1-yl)acetic acid 2-[2,5-dimethyl-1-(4-trifluoromethoxyphenyl)-1H-pyrrol-3-yl]-2-oxoethyl ester* (**7c**). IR (KBr, cm^-1^) 1670, 1695, 1770;^1^H-NMR: δ 1.96 (s, 3H), 2.23 (s, 3H), 4.86 (s, 2H), 5.22 (s, 2H), 6.27 (t, 1H, *J =* 5.4 Hz), 6.42 (d, 1H, *J =* 9.0 Hz), 6.49 (s, 1H), 7.46 (dd, 1H, *J =* 6.3 Hz, *J =* 2.1 Hz), 7.50 (d, 2H, *J =* 8.8 Hz), 7.57 (d, 2H, *J =* 8.8 Hz), 7.73 (dd, 1H, *J =* 5.1 Hz, *J =* 1.9 Hz); ^19^F-NMR: - 57.16 (F_3_CO); MS: *m/z* 449.2 (M+1)^+^; Anal. Calcd for C_22_H_19_F_3_N_2_O_5_: C 58.93; H 4.27; N 6.25. Found: C 58.82; H 4.24; N 6.16.

### 3.4. General method for synthesis of 2,5-dimethyl-3-(2-S-substituted 1-oxoethyl)-1-[4-(trifluoromethoxy)phenyl]-1H-pyrroles **8 a-c**

An appropriate thione (5 mmol) and potassium сarbonate (1.4 g, 10 mmol) were added to a solution of2-сhloro-1-{2,5-dimethyl-1-[4-(trifluoromethoxy)phenyl]-1*H*-pyrrol-3-yl}-ethanone (**5**, 1.66 g, 5 mmol) in DMF (50 mL). The reaction mixture was heated at 65-70 °C with stirring for 2 h, and then it was cooled and poured into water (100 mL). The precipitated solid was collected, dried and recrystallized from ethanol/DMF 4:1.

*1-[2,5-Dimethyl-1-(4-trifluoromethoxyphenyl)-1H-pyrrol-3-yl]-2-([1,3,4]thiadiazol-2-ylsulfanyl)-ethanone* (**8a**). ^1^H-NMR: δ 1.99 (s, 3H), 2.25 (s, 3H), 4.77 (s, 2H), 6.57 (s, 1H), 7.45 (d, 2H, *J =*7.6 Hz), 7.50 (d, 2H, *J =* 7.6 Hz), 9.50 (s,1H) ^19^F-NMR: - 57.50 (F_3_CO); MS: *m/z* 414.0 (M+1)^+^; Anal. Calcd. for C_17_H_14_F_3_N_2_O_2_S_2_: C 59.45; H 4.99; N 12.23 Found: C 59.58; H 4.91; N 12.32.

*1-[2,5-Dimethyl-1-(4-trifluoromethoxyphenyl)-1H-pyrrol-3-yl]-2-(5-[1,2,4]triazol-1-ylmethyl-[1,3,4]-oxadiazol-2-ylsulfanyl)ethanone* (**8b**). ^1^H-NMR: δ 1.99 (s, 3H), 2.24 (s, 3H), 4.73 (s, 2H), 5.80 (s, 2H), 6.52 (s, 1H), 7.49 (d, 2H, *J =* 9.0 Hz), 7.52 (d, 2H, *J =* 9.0 Hz), 7.95 (s, 1H), 8.60 (s, 1H). ^19^F-NMR: - 57.47 (F_3_CO); MS: *m/z* 479.0 (M+1)^+^; Anal. Calcd. for C_20_H_17_F_3_N_6_O_3_S: C 50.21; H 3.58; N 17.56. Found: C 50.32; H 3.52; N 17.63.

*1-[2,5-Dimethyl-1-(4-trifluoromethoxyphenyl)-1H-pyrrol-3-yl]-2-(1-methyl-1H-tetrazol-5-ylsulfanyl)ethanone*** (8c).** ^1^H NMR (DMSO-d_6_): δ 1.99 (s, 3H), 2.24 (s, 3H), 4.00 (s, 3H), 4.75 (s, 2H), 6.54 (s, 1H), 7.47 (d, 2H, *J =* 9.0 Hz), 7.53 (d, 2H, *J =* 9.0 Hz). ^19^F NMR (DMSO-d_6_): - 57.47 (F_3_CO). MS: m/z 412.0 (M+1)^+^. Anal. Calcd. for C_17_H_16_F_3_N_5_O_2_S: C 49.63; H 3.92; N 17.02. Found: C 49.75; H 3.82; N 17.13.

### 3.5. General method for synthesis of 4-[2,5-dimethyl-1-(4-trifluoromethoxyphenyl)-1H-pyrrol-3-yl]thiazoles **9a-g**

A mixture of 2-сhloro-1-{2,5-dimethyl-*1-[4-(trifluoromethoxy)phenyl]-1H*-pyrrol-3-yl}-ethanone (**5**, 1.66 g, 5 mmol) and thioamide/thiourea (5 mmol) in ethanol (40 mL) was refluxed for 5 h. The solid separated on cooling was collected by filtration, dried and recrystallized from ethanol.

*4-[2,5-Dimethyl-1-(4-trifluoromethoxyphenyl)-1H-pyrrol-3-yl]-2-methylthiazole* (**9a**). ^1^H-NMR: δ 2.00 (s, 3H), 2.25 (s, 3H), 2.67 (s, 3H), 6.24 (s, 1H), 7.15 (s, 1H), 7.44 (d, 2H, *J =* 9.0 Hz), 7.50 (d, 2H, *J =* 9.0 Hz);^ 19^F-NMR: - 57.43 (F_3_CO); MS: *m/z* 353.0 (M+1)^+^; Anal. Calcd. for C_17_H_15_F_3_N_2_OS: C 57.95; H 4.29; N 7.95. Found: C 57.86; H 4.21; N 8.04.

*4-[2,5-Dimethyl-1-(4-trifluoromethoxyphenyl)-1H-pyrrol-3-yl]-2-thiophen-2-ylthiazole* (**9b**). ^1^H-NMR: δ 2.03 (s, 3H), 2.32 (s, 3H), 6.35 (s, 1H), 7.12 (t, 1H, *J =* 5.6 Hz), 7.20 (s, 1H), 7.42 (d, 2H, *J =* 8.7 Hz), 7.47 (d, 2H, *J =* 8.7 Hz), 7.70 (m, 2H);^ 19^F-NMR (DMSO-d_6_): - 57.69 (F_3_CO); MS: *m/z* 421.0 (M+1)^+^; Anal. Calcd. for C_20_H_15_F_3_N_2_OS_2_: C 57.13, H 3.60; N 6.66. Found: C 57.19; H 3.55; N 6.74.

*2-{4-[2,5-Dimethyl-1-(4-trifluoromethoxyphenyl)-1H-pyrrol-3-yl]thiazol-2-yl}-N-phenylacetamide* (**9c**). ^1^H-NMR: δ 1.99 (s, 3H), 2.24 (s, 3H), 4.25 (s, 2H), 6.28 (s, 1H), 7.03 (t, 1H, *J =*7.6 Hz), 7.31 (m,2H) 7.41 (s, 1H), 7.48 (d, 2H, *J =*9.0 Hz), 7.52 (m, 2H), 7.64 (d, 2H, *J =*9.0 Hz), 10.55 (s,1H); ^19^F-NMR: - 57.43 (F_3_CO). MS: *m/z* 472.0 (M+1)^+^; Anal. Calcd. for C_24_H_20_F_3_N_3_O_2_S: C 61.14, H 4.28; N 8.91. Found: C 61.29; H 4.21; N 8.84.

*4-[2,5-Dimethyl-1-(4-trifluoromethoxyphenyl)-1H-pyrrol-3-yl]thiazol-2-ylamine* (**9d**). ^1^H-NMR: δ 2.02 (s, 3H), 2.12 (s, 3H), 6.27 (s, 1H), 6.49 (s, 1H), 7.43 (d, 2H, *J =* 8.7 Hz), 7.48 (d, 2H, *J =* 8.7 Hz), 9.10 (br s, 2H). ^19^F-NMR: - 57.70 (F_3_CO). MS: m/z 354.0 (M+1)^+^; Anal. Calcd. for C_16_H_14_F_3_N_3_OS: C 54.38; H 3.99; N 11.89. Found: C 54.46; H 4.07; N 11.80.

*{4-[2,5-Dimethyl-1-(4-trifluoromethoxyphenyl)-1H-pyrrol-3-yl]thiazol-2-yl}methylamine* (**9e**). ^1^H- NMR: δ 1.90 (s, 3H), 2.25 (s, 3H), 3.00 (s, 3H), 6.24 (s, 1H), 6.65 (s, 1H), 7.45 (d, 2H, *J =* 9.0 Hz), 7.51 (d, 2H, *J =* 9.0 Hz), 9.34 (br s, 1H); ^19^F-NMR: - 57.43 (F_3_CO). MS: *m/z* 368.0 (M+1)^+^; Anal. Calcd. for C_17_H_16_F_3_N_3_OS: C 55.58; H 4.39; N 11.44. Found: C 55.67; H 4.31; N 11.50.

*{4-[2,5-Dimethyl-1-(4-trifluoromethoxyphenyl)-1H-pyrrol-3-yl]thiazol-2-yl}-(2-methoxyethyl)amine* (**9f**). ^1^H-NMR: δ 1.99 (s, 3H), 2.24 (s, 3H), 3.29 (s, 3H), 3.43 (t, 2H, *J =* 5.4 Hz), 3.52 (t, 2H, *J =* 5.4 Hz), 6.12 (s, 1H), 6.23 (s, 1H), 7.40 (d, 2H, *J =* 9.0 Hz), 7.48 (d, 2H, *J =*9.0 Hz); ^19^F-NMR: - 57.70 (F_3_CO); MS: *m/z* 412.0 (M+1)^+^; Anal. Calcd for C_19_H_20_F_3_N_3_O_2_S: C 55.47; H 4.80; N 10.21. Found: C 55.58; H 4.79; N 10.09.

*{4-[2,5-Dimethyl-1-(4-trifluoromethoxyphenyl)-1H-pyrrol-3-yl]thiazol-2-yl}-(tetrahydrofuran-2-yl-methyl)amine* (**9g**). ^1^H-NMR: δ 1.62 (m, 1H), 1.73 (m,2H), 1.85 (m, 1H), 1.96 (s, 3H), 2.23 (s, 3H), 3.25 (s,2H), 3.64 (m, 1H), 3.77 (m, 1H), 4.03 (m, 1H), 6.13 (s, 1H), 6.27 (s, 1H), 7.41 (d, 2H, *J =* 8.7 Hz), 7.47 (d, 2H, *J =* 8.7 Hz), 8.56 (m, 1H); ^19^F-NMR: - 57.47 (F_3_CO); MS: *m/z* 438.0 (M+1)^+^; Anal. Calcd for C_21_H_22_F_3_N_3_O_2_S: C 57.66; H 4.5.07; N 9.60. Found: C 57.79; H 5.09; N 9.49.

## 4. Conclusions

In summary, we developed a successful chain heterocyclization strategy for the synthesis of A‑B‑C fluoroheterocyclic systems with pyrrole as the central unit B. These compounds were prepared in four steps starting from commercially available 4-trifluoromethoxyaniline. The pyrrole ring was added in one step using Paal-Knorr method, and the third thiazole ring was added in three steps using chloroacylation with cloroacetonitrile followed by heterocyclization with thioamide/thiourea. We are currently evaluating biological profiles of these new fluorinated heterocyclic systems.
